# Gestational exposure to the cannabinoid WIN 55,212-2 and its effect on the innate intestinal immune response

**DOI:** 10.1038/s41598-019-56653-y

**Published:** 2019-12-30

**Authors:** Rosalía Hernández-Cervantes, Armando Pérez-Torres, Óscar Prospéro-García, Jorge Morales Montor

**Affiliations:** 1 Departamento de Inmunología, Instituto de Investigaciones Biomédicas, Universidad Nacional Autónoma de México, Mexico City, Mexico; 2 Departamento de Biología Celular y Tisular, Facultad de Medicina, Universidad Nacional Autónoma de México, Mexico City, Mexico; 3 Grupo de Neurociencias, Laboratorio de Cannabinoides, Departamento de Fisiología, Facultad de Medicina, Universidad Nacional Autónoma de México, Mexico City, Mexico

**Keywords:** Interleukins, Neurophysiology

## Abstract

The consequences of marijuana consumption during pregnancy and its effects on the function of the immune system have been little studied. Marijuana is one of the most consumed recreational drugs among pregnant women, and it is known that gestational exposure to marijuana can have serious effects on the offspring after birth. In this study, we challenged the immune system of Wistar rats by infecting them with the parasitic nematode *Trichinella spiralis*. A treatment group of these animals was prenatally exposed to the cannabinoid WIN 55,212-2; a control group was not exposed. At 5 days of infection, the treated animals were less effective in eliminating intestinal parasites; moreover, this effect was correlated with a deficiency in mucus production, lower recruitment of eosinophils in the duodenum, and a reduced percentage of Tγδ and NK cells. In conclusion, the gestational administration of the synthetic cannabinoid WIN 55,212-2 induces lasting changes to the function of the immune system against infection with *T. spiralis* in male Wistar rats, making them more susceptible to infection.

## Introduction

Cannabis is currently the most cultivated, produced, trafficked and consumed drug worldwide. Approximately 3.8% of the world’s population consumes it; this is followed by cocaine, hallucinogens such as LSD and ecstasy, inhalants, methamphetamines, and heroin^1^. For every two men who consume marijuana, one woman does so^2^, either for recreational or medicinal purposes. In the United States, marijuana is the most consumed drug by women of reproductive age, followed by psychotherapeutic drugs such as codeine, inhalants, methamphetamines, cocaine, and heroin^1^. Induced appetite is included among the medicinal purposes of marijuana in patients with HIV and cancer, and it is also used to lessen nausea and vomiting in people undergoing chemotherapy. Another medicinal purpose relevant to this study is reducing nausea and vomiting in pregnant women during the first trimester of gestation^1^.

Pregnancy involves critical stages of development; therefore, the fetus may be compromised by exposure to external agents. In this context, it is known that Δ9-tetrahydrocannabinol (THC), a psychotropic cannabinoid found in marijuana, can traverse the placental barrier into the amniotic fluid and even into fetuses^3^. Previous studies indicate that gestational exposure to marijuana induces lasting effects, even after birth, in animal and human models. In humans, gestational exposure to marijuana may reduce weight and size in newborn infants^4^. Abnormalities in the nervous system have also been reported in babies born from women who smoked marijuana before and during pregnancy. The babies exposed to marijuana before birth presented increased tremors and overjumps^5,6^ and lesser habituation to visual stimuli^6^. The children of marijuana-consuming mothers are also more likely consume it during their adult life^7^.

Adult mice that have been gestationally exposed to THC or cannabidiol show altered reproductive parameters that are made evident by smaller testicles. Such an effect is induced by elevated levels of luteinizing hormone during and after sexual maturation. Further, both THC and cannabidiol interfere with testicular cholesterol esterase, an enzyme involved in testosterone production^8^. Should THC be administered during the last week of gestation, the copulative behavior of adult male mice becomes inhibited. Moreover, they show decreased secretion of prolactin, absent ejaculation, and increased mount latency. On the other hand, female mice exposed to THC during the first days after birth show decreased levels of luteinizing hormone, irregular estrus cycles, and diminished lordosis behavior^8,9^. In addition, gestational exposure to the synthetic cannabinoid HU-210 has been associated with a decreased response of the hypothalamus-pituitary-adrenal axis and with increased levels of corticosterone in adult rats exposed during the last days of gestation^10^.

**Figure 1:**
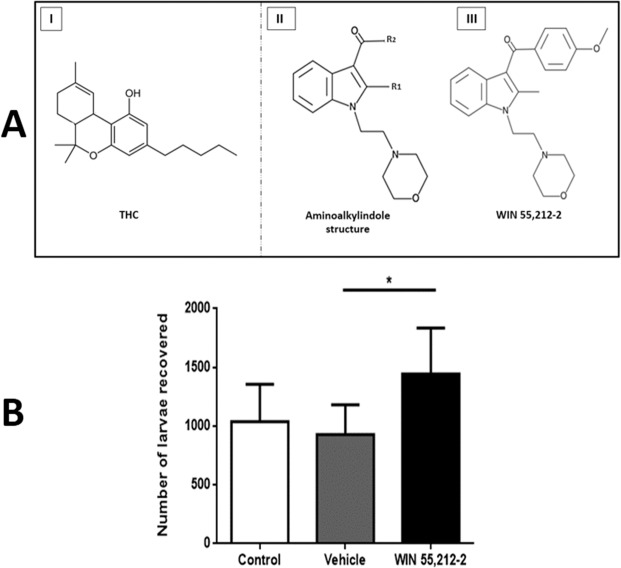
(**A**) Structure of the main cannabinoid of the Cannabis sativa plant, tetrahydrocannabinol, THC (I). General structure of aminoalkylindole compounds (II), derived from pravadoline, which have the same biological effects as THC, with WIN 55,212-2 being the most representative compound (III). (**B**) Parasitic load in the duodenum of the three experimental groups. Animals were infected with 2000 muscular larvae by intragastric route and sacrificed after 5 days post-infection. The larvae were recovered from the gut of infected animals. Data are expressed as mean ± SD. One-way ANOVA test and post hoc Tukey multiple comparisons test was performed. *p < 0.05; n = 6 for each group.

WIN 55,212-2 is another synthetic cannabinoid with reported long-lasting effects, such as long-term alteration of the endocannabinoid system (ECS) in brain areas involved with learning-memory, motor activity and emotional behavior^11–15^. WIN 55,212-2, an aminoalquilindol compound derived from pravadoline, possesses a biological activity similar to THC, although with a different chemical structure (Fig. 1A)^16^. This synthetic cannabinoid has a greater affinity for cannabinoid receptors than THC (e.g. it has a ki = 1.89 nM for the CB1 receptor and ki = 0.28 nM for the CB2 receptor). In comparison, THC has an affinity of 40.7 nM and 36.4 nM for the CB1 and CB2 receptors, respectively^17^. Therefore, lower amounts of this compound are needed to produce the same effects as THC, since only 0.5 mg/kg bw of WIN 55,212-2 can have a moderate effect in rats, whereas 10 times more THC is needed (5 mg/kg bw) to obtain the same effect.

The long-term effects of gestational exposure to cannabinoid compounds on the function of the immune system have been little studied. However, it is known that mice prenatally exposed to THC on gestational day 16 display thymic atrophy and marked alterations in T cell subpopulations, such as a decreased number of CD8^+^, CD4^+^CD8^+^ and CD4^-^CD8^-^ T cell subsets. Such an effect is dependent upon the CB1 and CB2 receptors^18^. Also, pregnant mice exposed to THC displayed a persistent effect on the postnatal immune response. Adult mice inoculated with the HIV-1 proteins p17/P24/gp120 showed a decreased level of specific antibodies as well as a decreased proliferation of T lymphocytes^18^.

The immune system has the important function of defending the host from foreign agents or pathogens and, depending on their nature, making use of innate and/or adaptive immunity mechanisms to eliminate them. The use of cannabis increases susceptibility to infectious diseases, regardless of whether they are caused by bacteria, viruses, parasites or, to a lesser degree, fungi^19^. The long-term effects of the gestational use of cannabis on the immune system during infectious diseases have not been studied so far.

*Trichinella spiralis* is an intracellular nematode that colonizes the striated muscles of infected mammals. This zoonosis, known as trichinosis, is commonly caused by the consumption of raw or undercooked meat from infected animals^20^. The establishment of the parasite in the duodenum represents the acute, or enteric, phase characterized by goblet cell (GC) hyperplasia, increased mucin and intestinal trefoil factor expression, and an inflammatory infiltration in the lamina propria^21^. At this stage, the intestinal inflammatory infiltrate is composed of lymphocytes, mast cells and eosinophils recruited to the intestinal Peyer patches and solitary lymphatic nodes^21^. Mastocitosis in the intestinal mucosa is also a typical feature of infection with *T. spiralis*^21^. Such activation of mast cells, followed by the secretion of its mediators, has been involved in the expulsion of parasites. It is important to highlight that the combination of *T. spiralis* infection and marijuana use is very common in the gestational period in many countries.

The aim of this study was to evaluate the long-term effects on the function of the immune system in animals exposed prenatally to the THC analog WIN 55,212-2. Further, an acute infection with the parasite *T. spiralis* was used as an antigenic challenge, because it is a cosmopolitan nematode acquired by consuming raw or badly cooked meat infested with larvae, whose development during the intestinal phase is critical for the course of infection.

## Results

### General effects of perinatal WIN 55,212-2 exposure on reproductive parameters

There was no significant difference in weight gain (%) during pregnancy in the experimental groups (control 34.1 ± 2.4, vehicle 24.7 ± 15, WIN 55,212-2 30.2 ± 1.6). Neither group was altered in terms of the gestational period (days) (control 21.5 ± 0.7, vehicle 21 ± 0, WIN 55,212-2 21 ± 0), the number of offspring (control 10.67 ± 1.52, vehicle 10.25 ± 2.98, WIN 55,212-2 7.8 ± 2.94) or the post-gestational mortality rate (control 3.03 ± 5.24, vehicle 2.77 ± 5.55, WIN 55,212-2 1.66 ± 3.72) (Table 1).

**Table 1:** Reproductive parameters.

Group	Maternal weight gain (%)	Gestation period (days)	Number of offspring	Postnatal mortality (%)	Weight gain of rat offspring (grams)				
					0	7	14	30	60
Control	34.1 ± 2.4	21.5 ± 0.7	10.7 ± 1.5	3 ± 1	5.8	13.9	25.9	96	216.3
Vehicle	24.7 ± 1.5	21 ± 0	10.3 ± 2.98	2.77 ± 1.5	6.2	14.4	28.3	95.8	207.9
WIN 55,212-2	30.2 ± 1.6	21 ± 0	7.8 ± 2.94	1.66 ± 3.7	6.4	14.5	26.2	92.3	202.6

Different data were recorded during and after pregnancy, such as weight gain of the pregnant rats, the gestation period (days), the number of offspring, postnatal mortality and the weight of the offspring at different days.

The results are expressed as the mean ± SD of three experiments by quintuplicate (n = 15).

### Effects of perinatal WIN 55,212-2 exposure on parasitic load in adult rats

The animals treated with WIN 55,212-2 during gestation were infected with 2,000 muscular larvae of *T. spiralis* during adulthood. Five days after infection, the parasites that were not expelled were recovered from the gut of the infected animals and counted (control 1038 ± 319, vehicle 927 ± 255, WIN 55,212-2 1443 ± 390) (Fig. 1B). The WIN55,212-2-treated group was less effective in expelling *T. spiralis* larvae at 5 days post-infection in comparison to the infected vehicle group.

### Perinatal exposure to WIN 55,212-2 affects goblet cell morphology and eosinophil recruitment

**Figure 2:**
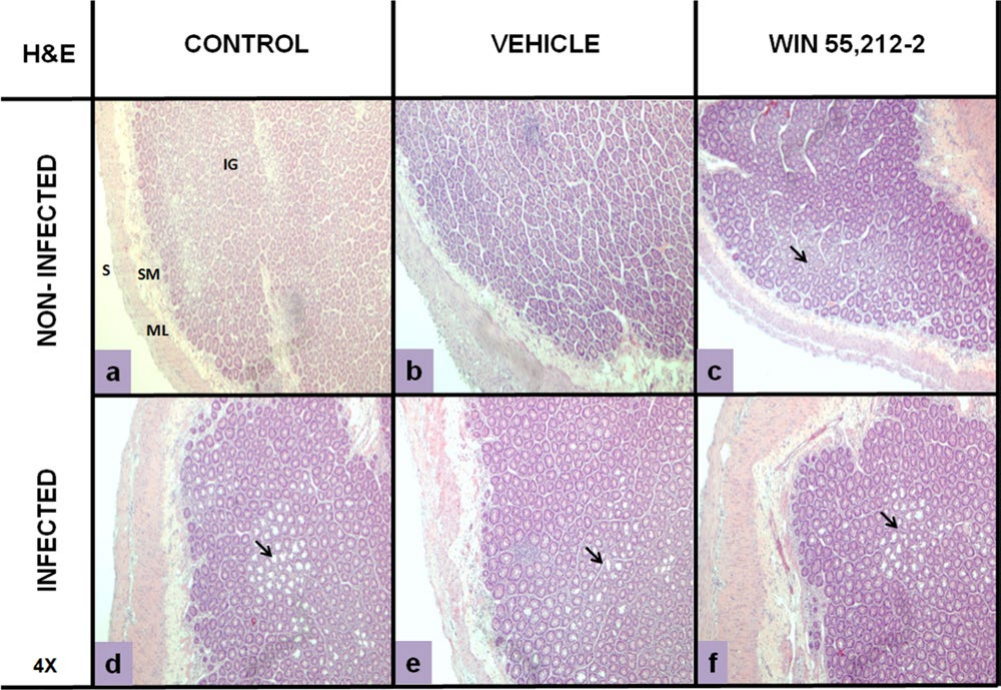
Morphology of duodenum. Hematoxylin and eosin staining (H&E; 4×) in non-infected (a,b,c) and infected (d,e,f) animals. IG: intestinal glands; ML: mucosal layer; S: serosa; SL: submucosal layer. Black arrows show damage at intestinal-gland level.

**Figure 3:**
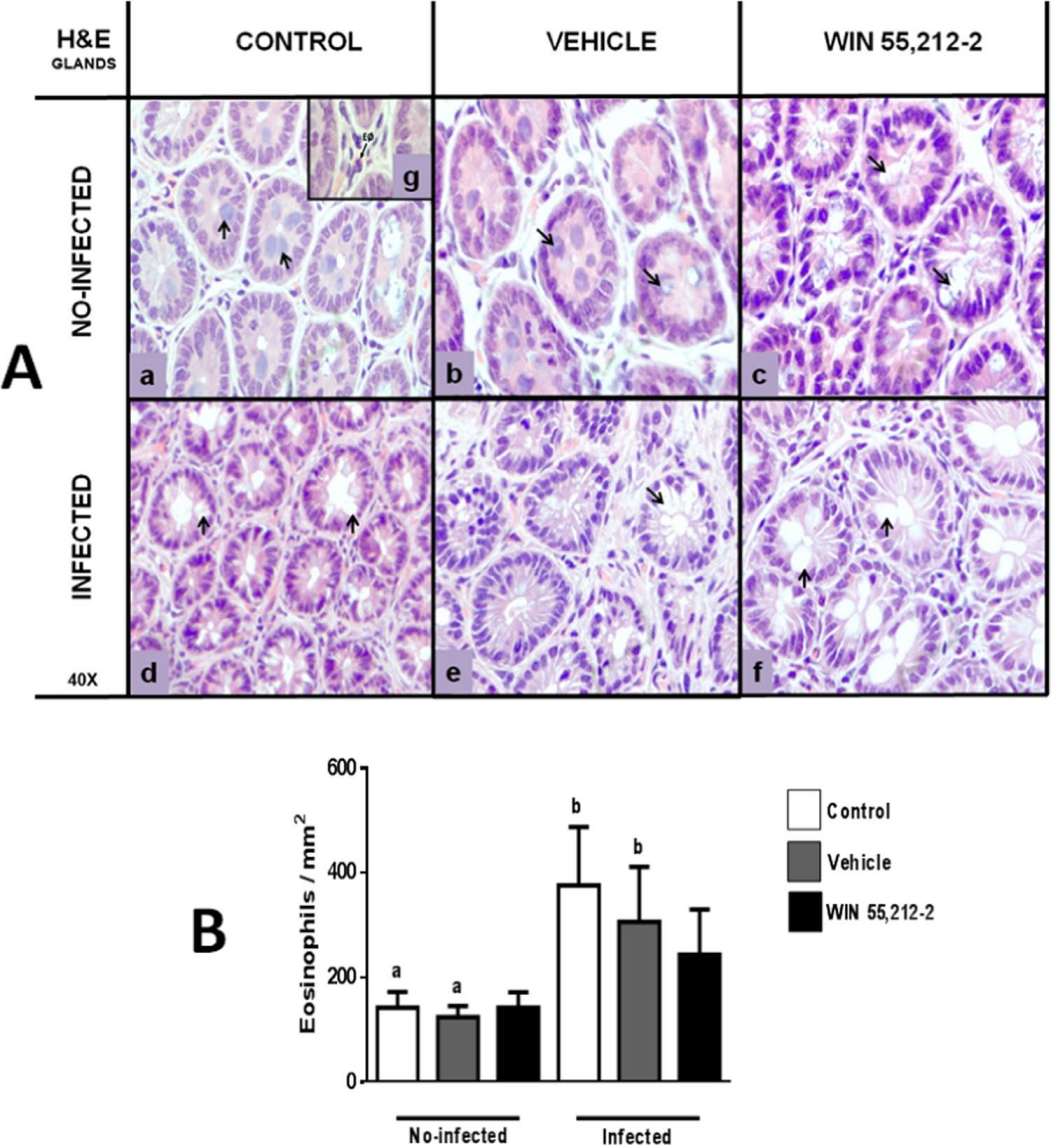
Morphology of glands in the duodenum. (**A**) Representative gland images from control (a,d), vehicle (b,e) and WIN 55,212-2 (c,f) experimental groups, in either non-infected (a,b,c) or infected (d,e,f) groups. Black arrows show goblet cells. The square (g) shows a representative eosinophil with ematoxylin and eosin stain (H&E; 40X). (**B**) Quantification of eosinophils. Data are expressed as mean ± *SD*. Two-way ANOVA test and post hoc Tukey multiple comparisons test were performed. Differences among treatments are shown with bars. Letters above each column indicate statistical differences among groups due to infection: a, no significant difference compared with a; b, no significant difference compared with b; *p* < 0.05 for a compared to b, or vice versa; *n* = 6 for each group.

**Figure 4:**
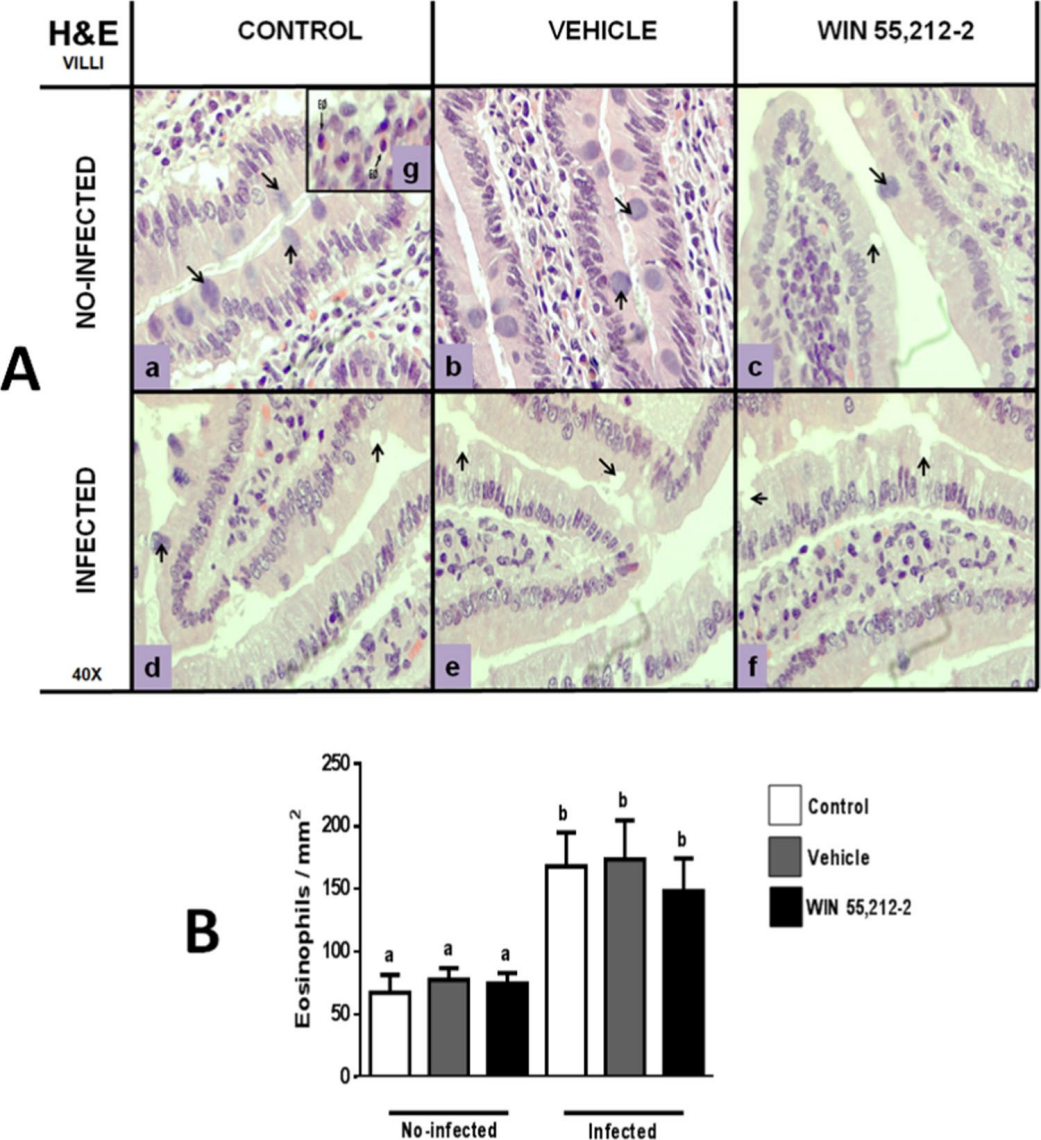
Morphology of villi in the duodenum. (**A**) Representative villi images from control (a,d), vehicle (b,e) and WIN 55,212-2 (c,f) experimental groups, in either non-infected (a,b,c) or infected (d,e,f) groups. Black arrows show goblet cells. The square (g) shows a representative eosinophil with hematoxylin and eosin stain (H&E; 40X). (**B**) Eosinophil quantification. Data are expressed as mean ± *SD*. Two-way ANOVA test and post hoc Tukey multiple comparisons test were performed. Differences among treatments are shown with bars. Letters above each column indicate statistical differences among groups due to infection: a, no significant difference compared with a; b, no significant difference compared with b; *p* < 0.05 for a compared to b, or vice versa; *n* = 6 for each group.

The histological analysis of duodenum morphology showed that the WIN 55,212-2-treated group presented white areas at the level of intestinal glands (Fig. 2c) that were similar to those found in all infected groups (Fig. 2d–f). Hematoxylin and eosin (H&E) staining of duodenum samples revealed that the white areas corresponded to GCs in both glands and villi. These white areas were different in the treated group in terms of coloration and looked empty (Figs. 3A,c and 4A,c) in both glands (Fig. 3A,d,e,f) and villi (Fig. 4A,d–f), similar to all infected groups.

**Figure 5:**
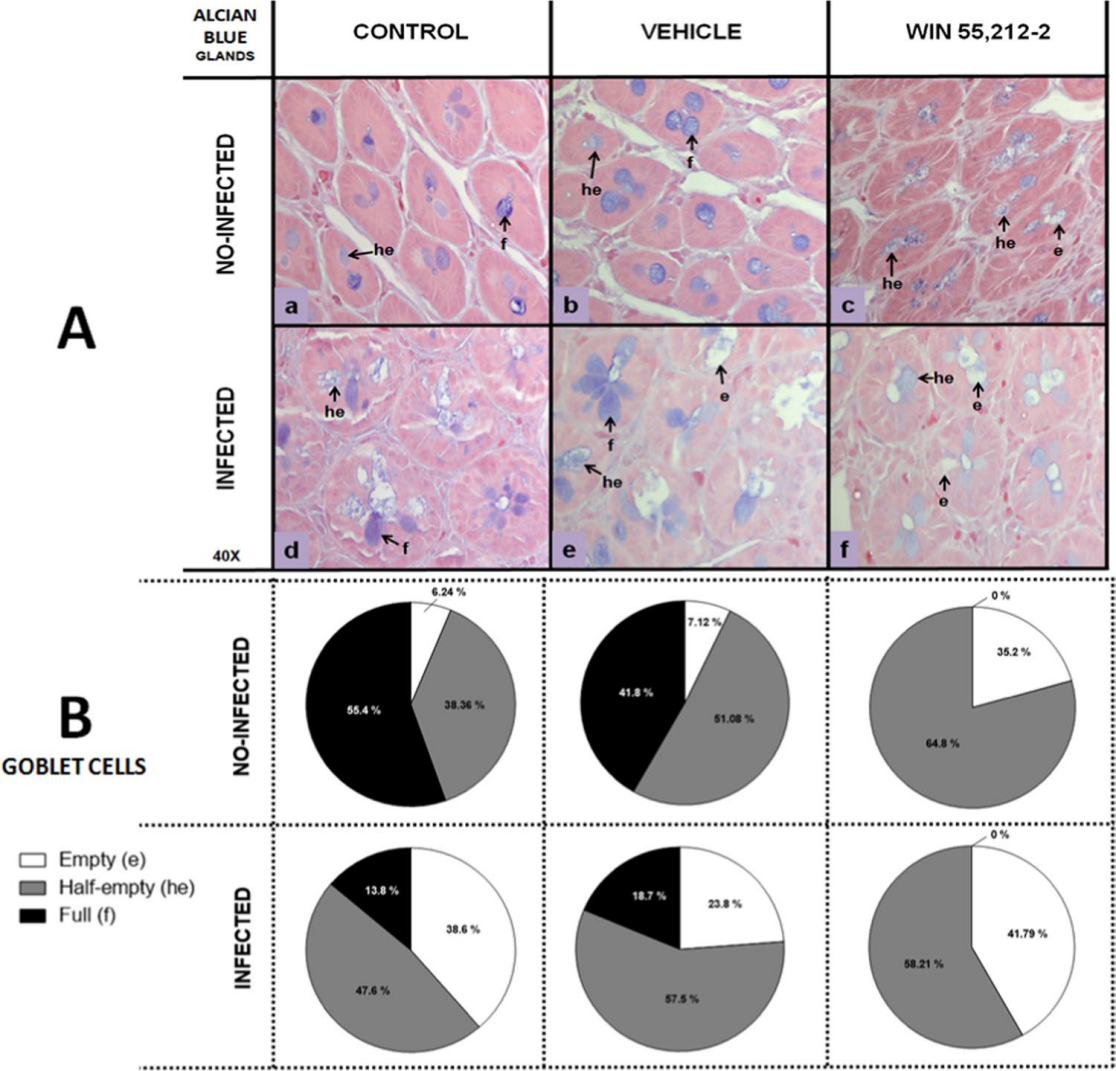
Goblet cells in glands of the duodenum. (**A**) Representative goblet cell images in the glands of the duodenum in control (a,d), vehicle (b,e) and WIN 55,212-2 (c,f) experimental groups, in either non-infected (a,b,c) or infected (d,e,f) groups. Goblet cells are stained blue. (**B**) Goblet cell quantification according to filling degree (e: empty, he: half-empty, f: full), with Alcian blue staining (40X). Data are expressed as mean percentage ± *SD*; *n* = 6 for each group.

**Figure 6:**
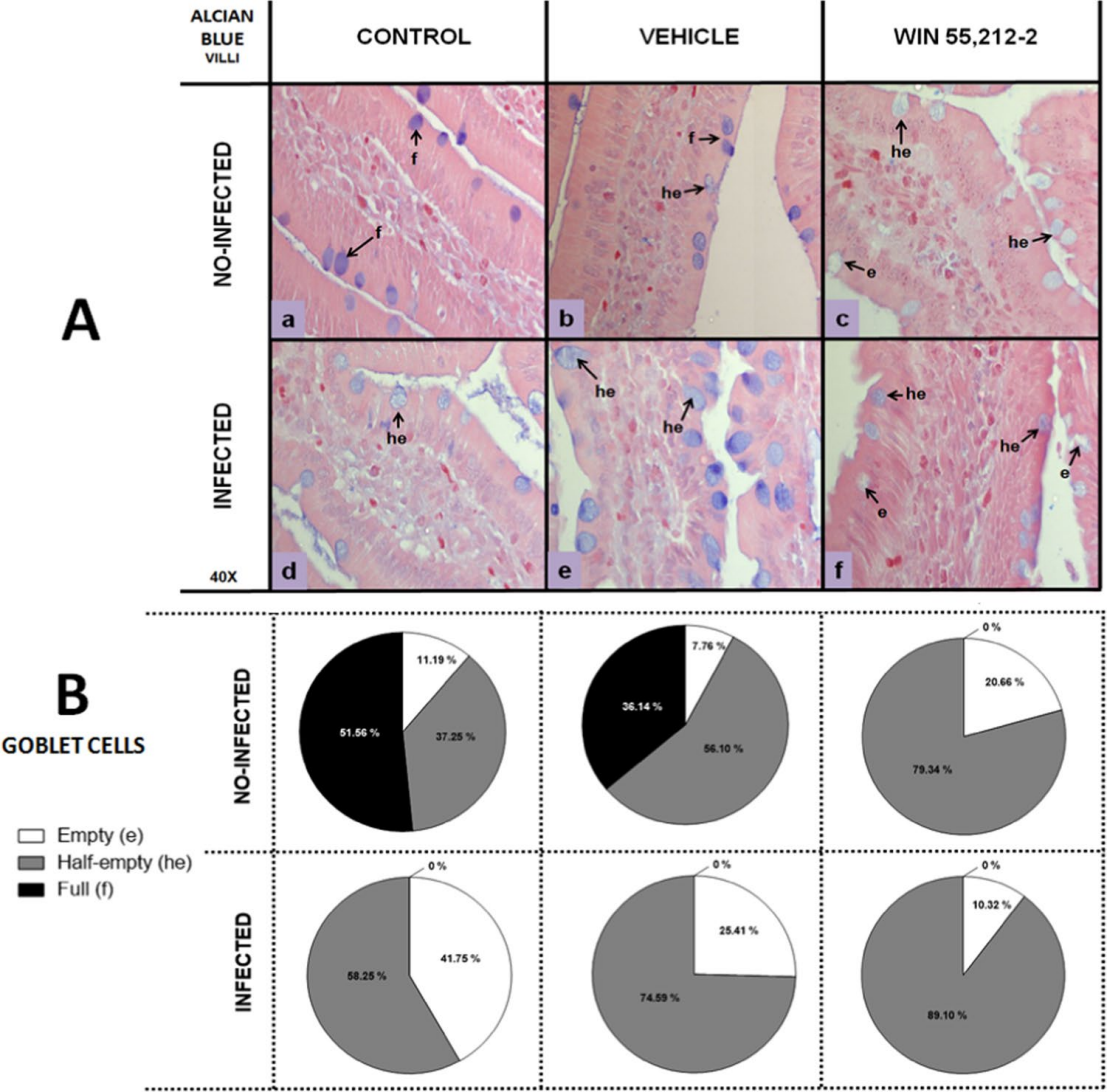
Goblet cells in villi of the duodenum. (**A**) Representative goblet cell images in the villi of the duodenum in control (a,d), vehicle (b,e) and WIN 55,212-2 (c, f) experimental groups, in either non-infected (a,b,c) or infected (d,e,f) groups. Goblet cells are stained blue. (**B**) Goblet cell quantification according to filling degree (e: empty, he: half-empty, f: full), with Alcian blue staining (40X). Data are expressed as mean percentage ± *SD*; *n* = 6 for each group.

Based on past observations, it was decided to perform an Alcian blue staining, which dyes the GCs in blue color. The results were similar to H&E staining. The WIN 55,212-2-treated group presented deficient GC filling in both glands (Fig. 5A,c) and villi (Fig. 6A,c), in a similar manner to the infected groups (Figs. 5A and 6A, d, e, f). The GCs of control and vehicle groups (Figs. 5A and 6A,a,b) looked different when compared against the WIN 55,212-2-treated group (Figs. 5A and 6A,c). Therefore, we designed a classification system based on the GCs’ coloration. The rounded cells in the center of a gland, or those in the periphery of a villus, were taken into account and divided into three categories based on color intensity: first, empty GCs (e), whose coloration was null or very tenuous; second, medium-empty GCs (he), whose content had approximately 50% of maximum coloration of the full GCs; and third, full GCs (f), whose coloration was intense. The percentage of observed GCs in glands and villi is found in Figs. 5B and 6B, respectively.

Regarding the glands, control and vehicle groups had a low percentage of empty GCs (6.24% and 7.12%, respectively). A larger proportion of GCs was half-empty or full (control 38.36% half-empty and 55.4% full; vehicle 51.08% half-empty and 41.8% full). Further, the WIN 55,212-2-treated group did not show full GCs and had a greater proportion of empty GCs (64.8%) (Fig. 5A,c). In the infected group, the percentages of empty and half-empty GCs in the control and vehicle groups were increased (control 38.6% empty and 47.6% half-empty; vehicle 23.8% empty and 57.5% half-empty), whereas in the WIN 55,212-2-treated group, the percentage of empty GCs was increased in comparison with the non-infected treated group (41.79% *vs*. 35.2%, respectively).

Similar results were observed in the villi, revealing that non-infected control and vehicle groups had lower percentages of empty GCs (control 11.19%, vehicle 7.76%), whereas the WIN 55,212-2-treated group did not present full GCs, an observation that was repeated in all the infected groups (Fig. 6B).

Eosinophils were also quantified in H&E stains. Regarding the glands, no significant differences were observed between the non-infected (control 142.5 ± 30.20, vehicle 124.5 ± 21.45, WIN 55,212-2 142.71 ± 29.49) and infected groups (control 376.25 ± 111.35, vehicle 306 ± 105.96, WIN 55,212-2 244 ± 86.35). However, the infected control and vehicle groups displayed increased eosinophil recruitment. Further, there were no significant differences due to infection between the WIN 55,212-2-treated groups, meaning that the infected group was less effective in recruiting eosinophils to the intestinal glands (Fig. 3B).

There were no significant differences in the villi due to gestational treatment (control 67.4 ± 14.08, vehicle 77.5 ± 9.37, WIN 55,212-2 74.62 ± 8.41). However, there was a difference due to infection, with all the infected groups showing increased numbers of recruited eosinophils (control 168 ± 27, vehicle 173.6 ± 31.14, WIN 55,212-2 148.2 ± 26.24) (Fig. 4B).

### Perinatal WIN 55,212-2 exposure modifies the populations of NK and Tγδ cells in the duodenum

**Figure 7:**
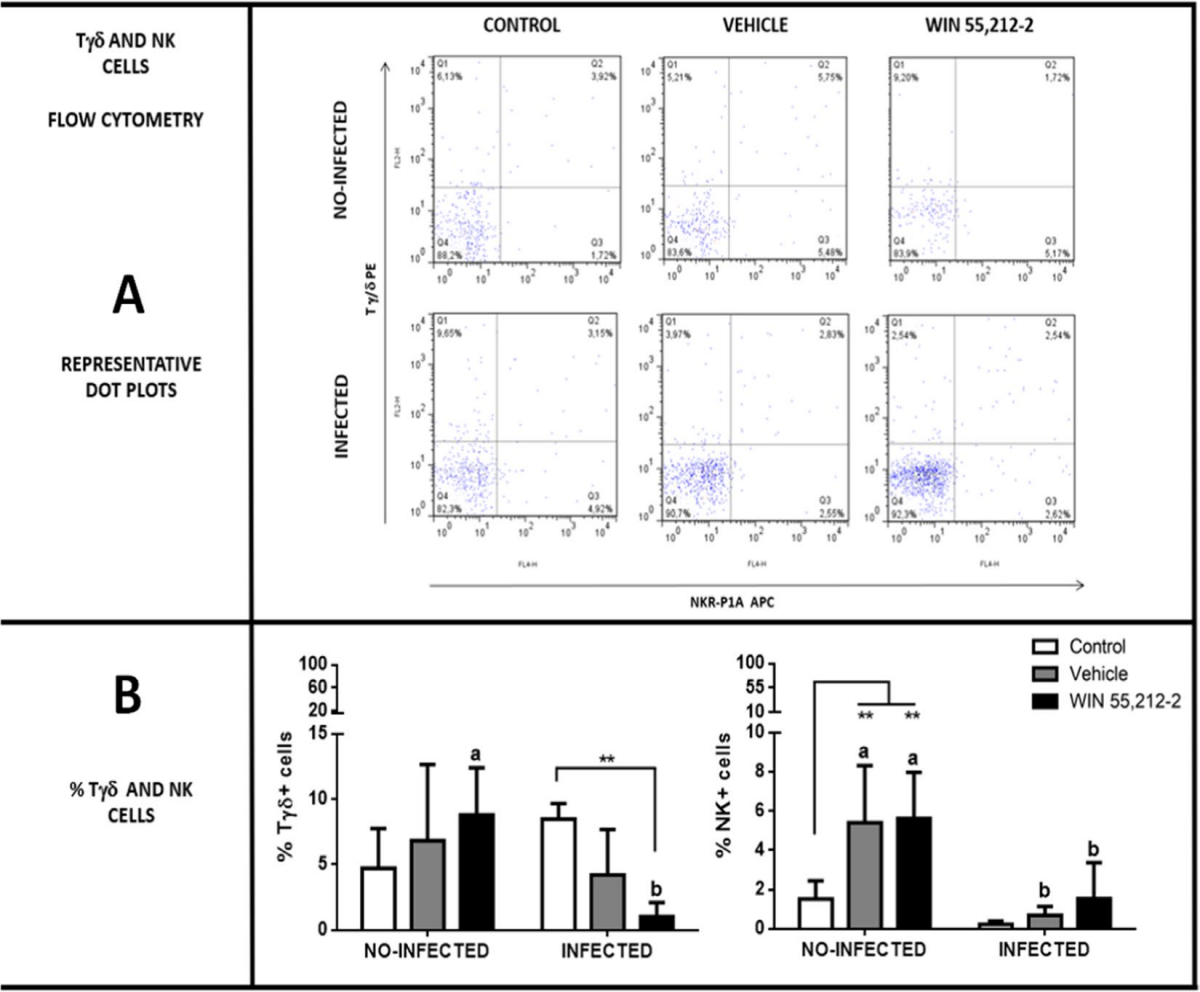
Percentage of NK and Tγδ cells in duodenum. (**A**) Representative dot plots in control, vehicle and WIN 55,512-2 groups of non-infected and infected groups. (**B**) Percentage of Tγδ and NK cells. Data are expressed as mean ± *SD*. Two-way ANOVA test and post hoc Tukey multiple comparisons test were performed. **p < 0.01; differences among treatments are shown with bars. Letters above each column indicate statistical differences among groups due to infection: a, no significant difference compared with a; b, no significant difference compared with b; *p* < 0.05 for a compared to b, or vice versa; *n* = 6 for each group.

The proportion of Tγδ and NK cells in duodenum samples was determined by flow cytometry. The gestational treatment with WIN 55,212-2 induced an increased presence of Tγδ cells (control 4.72 ± 3.03, vehicle 6.83 ± 5.81, WIN 55,212-2 8.35 ± 3.73). Interestingly, the number of these cells was reduced during the immune challenge (control 8.48 ± 1.18, vehicle 4.22 ± 3.47, WIN 55,212-2 1.02 ± 1.07) in comparison to the infected control group (Fig. 7B). The gestational treatment induced an increased number of NK cells in both the vehicle and WIN 55,212-2-treated groups (control 1.5 ± 0.92, vehicle 5.4 ± 2.91, WIN 55,212-2 5.61 ± 2.55). However, the number of NK cells decreased in all the groups during infection (control 0.23 ± 0.15, vehicle 0.69 ± 0.43, WIN 55,212-2 1.53 ± 1.83), although the increased number of NK cells was conserved in both the vehicle and the WIN 55,212-2-treated groups (Fig. 7B).

## Discussion

In the present study, a synthetic cannabinoid, WIN 55,212-2 (an analog of THC), was administered to Wistar rats during gestation, seeking to assess its effects on the function of the immune system in the adult offspring. The intent of this study is to extrapolate the findings to women who consume cannabinoids during their pregnancy, whether of natural or synthetic origin.

With the dose and management scheme of WIN 55,22-2 used in this study (0.5 mg/kg bw, from GD5 to GD16), no significant differences were reported in the parameters of reproduction, gestational period, number and weight of offspring or post-gestational mortality. The results are consistent with those from Harbison *et al*., where using doses greater than 1 mg/kg from GD1 to GD6 increased the frequency of fetal absorption and birth malformations^3^. Therefore, the results presented here are caused by the gestational treatment and/or the parasitic infection and not by discrepancies inherent to the birth of the exposed pups.

Because the gestational period is a critical stage of development, the harmful or beneficial effects of marijuana consumption cannot be generalized, mostly due to its dependence upon the dose used and the time and duration of exposure. Further, the potency of the consumed marijuana should be taken into account, especially because it has increased in recent decades and is now 6 to 7 times more potent compared with the marijuana available in 1970^20^. Furthermore, the THC content of marijuana has increased from 4% to 12% between 1995 and 2014^21^. Although marijuana consumption has long been described as a non-health risk, there are still serious issues related to behavioral and neurobiological disorders such as anxiety, depression, psychosis, cognitive deficits and addiction^22^.

The treatment with WIN 55,212-2 produced histological changes at the level of GCs. This is not the first time that an exogenous compound administered during gestation has produced morphological effects that are still visible after birth. This is also the case with the administration during gestation and lactation of BPA, an endocrine disruptor that increases the thickness of the uterine epithelium and the stroma, thus modifying the reproductive cycle of the exposed females^23^.

During infection with *T. spiralis*, the innate immune system is of utmost importance, because it will attempt to expel most of the parasites during the intestinal phase to avoid its systemic dispersion. No pharmacological treatment is available to eliminate the larvae at this stage. The GCs are responsible for producing mucus, and a *T. spiralis* infection produces a greater amount of mucus to isolate parasites from their biological niche. In addition to helminth infections, there is also an important recruitment of eosinophils, both being elements important for expelling parasites. In this study, it was observed that the WIN 55,212-2-treated group was less effective in expelling the parasites. There are three likely reasons for this ineffectiveness. First, it is not possible to isolate the parasites from their biological niche under a mucin deficiency. Second, the recruitment of eosinophils is much lower, and these cells play an important role in helminth infections. Third, the administration of the synthetic cannabinoid WIN 55,212-2 diminishes the number of Tγδ cells, which are found mainly in the mucosae and intervene in the intestinal immune response.

The cannabinoid receptors, together with endocannabinoids and enzymes for synthesis and degradation of the latter, form the ECS, which is already functional during the early development stages^24^. The so-called “double-hit hypothesis” states that prenatal exposure to cannabis, like a neurodevelopmental teratogen, produces a first blow to the ECS signaling pathway, which is compromised in such a way that a second hit (i.e. a postnatal stressor) precipitates the emergence of a specific phenotype^25^. In this work, the first blow is represented by gestational exposure to the analog synthetic cannabinoid of THC, WIN 55,212-2, while the infection with the parasite *T. spiralis* is the postnatal stressor. As a result, the animals exposed to WIN 55,212-2 during their gestational stage and infected during adulthood are more susceptible to greater parasitic burdens. Further, if the life cycle of the parasite is allowed to continue, these animals present a higher parasite load in the tissues, mainly in skeletal muscles, thus compromising their quality of life.

Although *T. spiralis* is a cosmopolitan nematode, parasitosis is poorly diagnosed during the intestinal stage, which is where treatment can be given to eliminate the larvae and thus prevent their systemic spread. The results from this study suggest that the innate immune system is altered by gestational exposure to a synthetic cannabinoid and triggered by an intestinal immunological challenge. It would be interesting to observe how the immune system behaves before a different antigenic challenge, or with the present one, in a systemic scenario, which would be closer to a clinical setting.

The gestational administration of cannabinoids for prenatal therapeutic purposes has been proposed in other cases. One such case is gastroschisis, a congenital abdominal wall defect characterized by a small orifice, generally located to the right of the navel, resulting in the herniation and permanent exposure of the bowel loops to amniotic fluid and its components during pregnancy. In this case, the administration of cannabidiol favors the anti-inflammatory effects on the intestinal loops and reduces iNOS levels^26^. Therefore, not all cannabinoids administered during pregnancy have negative effects.

**Figure 8:**
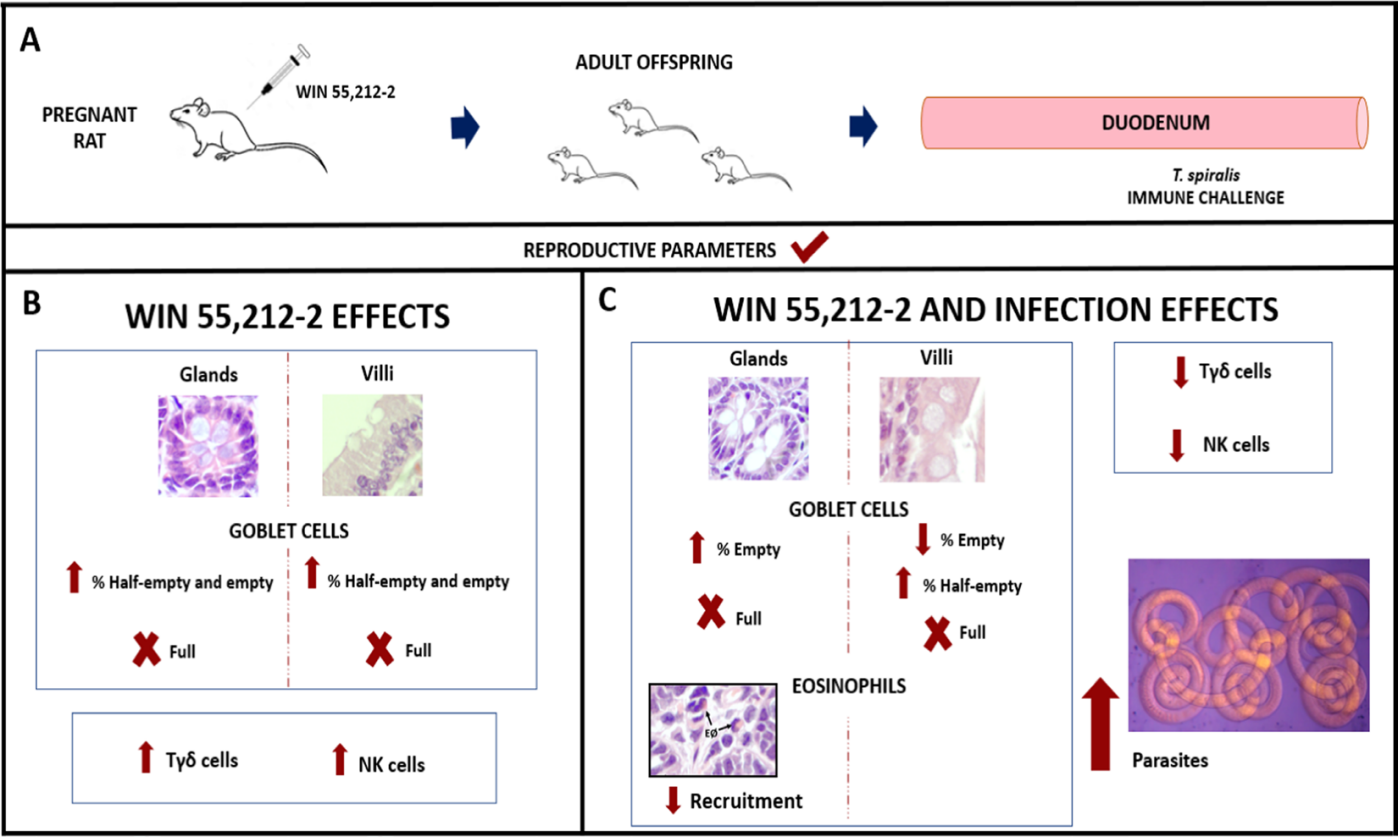
Summary of changes in the duodenum of adult rats prenatally exposed to WIN 55,212-2. Pregnant rats were treated with the synthetic cannabinoid WIN 55,212-2, and adult offspring were used for the posterior studies (**A**). The morphology of the duodenum and the percentage of Tγδ and NK cells were analyzed in the non-infected (**B**) and infected (**C**) adult offspring with *Trichinella spiralis*. Arrows indicate the increased or decreased values of the evaluated parameters, whereas “X” indicates their absence.

This study demonstrated that the gestational administration of the synthetic cannabinoid WIN 55,212-2 induces changes in the innate immunity of adult offspring that compromise its response to an acute infection with *T. spiralis*. In Fig. 8, it is a summary of our main findings, as a graphical conclusion.

## Methods

### Experimental design

The objective of the present study was to determine the long-term effects of gestational exposure to the synthetic cannabinoid WIN 55,212-2 on the function of the immune system in the exposed offspring, using an acute infection with *T. spiralis* as an antigenic challenge. The cannabinoid was administered to pregnant rats, and the immune system of the offspring was challenged once they reached adulthood.

### Ethics statement

Animal care and experimentation practice at the Instituto de Investigaciones Biomédicas are frequently evaluated by the Institute’s Animal Care and Use Committee, adhering to official Mexican regulations (NOM-062-ZOO-1999). Mexican regulations are in strict accordance with the recommendations stated in the Guide for the Care and Use of Laboratory Animals of the National Institutes of Health (NIH) of the USA and the Weatherall report to ensure compliance with established international regulations and guidelines. The protocol was approved by the Committee on the Ethics of Animal Experiments of the Instituto de Investigaciones Biomédicas (Permit Number 2017-00056).

### Animals and treatment

Nulliparous female Wistar rats (*Rattus norvegicus*), weighing 200–250 g, were housed at constant room temperature (20 ± 1 °C) and humidity (60%) with light/dark cycle of 12 h/day (6:00 to 18:00 h light) and food and water available *ad libitum*. The females in the proestrus phase were placed with a male for mating. Vaginal smears were taken the following morning at 09:00 h, where the presence of sperm cells was accepted as a probable sign of pregnancy. This day was designated as gestational day zero (GD0).

Pregnant rats were treated from gestational day 5 (GD5) to gestational day 16 (GD16) with doses of 0.5 mg/kg bw/day WIN 55,212-2 (Cayman Chemical, Michigan, USA). This dose was chosen based on a previous study^15^, in which it was determined that prolonged prenatal exposure to higher doses (1 mg/kg) affected reproduction parameters such as dam and pup weight gain and litter size at birth^15^. The drug was suspended in DMSO (Sigma*-*Aldrich, México) and PBS pH 7.2 (in a 1:2 relation), according to the manufacturer’s protocol, and injected subcutaneously (SC). The vehicle group was injected SC with DMSO/PBS pH 7.2 (1:2), whereas the control group did not receive any treatment.

After weaning, the offspring were sexed, and only males were used for the present study. Three to four animals receiving the same treatment were housed per cage.

### Experimental groups

Animals were organized into six groups of six animals each: Non-infected animals consisted of three groups: In the non-infected control group, pregnant rats did not receive any treatment. In the non-infected vehicle group, pregnant rats were treated with a mix of DMSO and PBS 1X, pH 7.2 (1:2) from DG0 to DG16. In the non-infected group treated with WIN 55,212-2, pregnant rats received 0.5 mg/kg wb/day from DG0 to DG16. Infected animals also consisted of three groups: In the infected control group, pregnant rats did not receive any treatment, but adult offspring were infected with 2000 muscular larvae of *T. spiralis*. In the infected vehicle group, pregnant rats were treated with a mix of DMSO and PBS 1X, pH 7.2 (1:2) from DG0 to DG16, and adult offspring were infected with 2000 muscular larvae of *T. spiralis*. In the infected group treated with WIN 55,212-2, pregnant rats received 0.5 mg/kg bw/day from DG0 to DG16, and adult offspring were infected with 2000 muscular larvae of *T. spiralis*.

### Infection and recuperation of parasites

*T. spiralis* (ISS 406) has been maintained by serial passage infections in Wistar rats in the laboratory used for the present study. The infective-stage nematodes (muscular larvae) were recovered from experimentally infected rats after 30 days post-infection. The infected muscle was digested using a standard pepsin-hydrochloric acid digestion method. Larvae were recovered and counted under a stereoscopic microscope (Bausch & Lomb).

At 12 weeks of age (adult age), six male rats per group were infected with 2000 muscular larvae by intragastric route (infected groups) and were euthanized at 5 days post-infection. The adult worms were recovered from the small intestine by dissecting longitudinally and washing the tissue in PBS 1X. Afterward, the intestinal tissue was sectioned into small pieces and incubated in PBS 1X for 3 hours at 37 °C. Following incubation, the parasites were collected and counted using a stereoscopic microscope (Bausch & Lomb).

### Duodenal histology

Fresh duodenum samples from each animal were fixed in 4% paraformaldehyde (J. T. Baker, México), dehydrated and embedded in paraffin. Cross-sections of 6 µm thickness were obtained and stained with H&E to observe the general morphology of the tissue and identify the eosinophils. Alcian blue staining was used to detect the GCs. The sections were analyzed under a light microscope at 4X and 40X magnification. The number of eosinophils was counted through several microscope fields, equivalent to a 1 mm^2^ area, and in triplicate for each animal.

### Flow cytometry

Approximately 1 cm of the duodenum was collected from each rat. The samples were washed with PBS 1X, finely cut and incubated for 30 minutes in digestion medium (RPMI 1640, Sigma-Aldrich, St. Louis, MO), 1 mg/ml DNase (Roche Applied Science, Mannheim, Germany) and 50 U/ml type IV collagenase (Sigma-Aldrich, St. Louis, MO). After 30 minutes at 37 °C, the digestion was stopped by adding medium with FBS and mesh disaggregation. The cell suspensions were centrifuged, and the pellets obtained were resuspended in FACS buffer (PBS, 2% FBS, 0.02% NaN_3_). Approximately 1 × 10^6^ cells were incubated for 20 minutes at 4 °C, washed and stained. The antibodies APC-conjugated anti-NKR P1A (Invitrogen, Carlsbad, CA) and PE-conjugated anti-TCR γδ (BioLegend, San Diego, CA) were used to identify the NK and Tγδ cell populations. The samples were processed in a FACS Calibur flow cytometer (BD Biosciences), and the obtained data were analyzed using the FlowJo software (Treestar Inc.)

### Statistical analysis

The general experimental design considered two independent variables: perinatal synthetic cannabinoid treatment (two levels: Yes or No) and infection (Yes or No). Data from two independent experiments (*n* = 6) were charted as mean ± standard deviation and analyzed with Prism 6® software (GraphPad Software Inc.). For the parasitic load, a one-way ANOVA was performed, while for the other experiments, a two-way ANOVA was performed, followed by a Tukey post-hoc test. Differences were considered significant when *p* < 0.05.

## Data Availability

The data sets generated and analyzed during the current study are available from the corresponding author on reasonable request.
